# Signal detectability and boldness are not the same: the function of defensive coloration in nudibranchs is distance-dependent

**DOI:** 10.1098/rspb.2023.1160

**Published:** 2023-07-26

**Authors:** Cedric P. van den Berg, John A. Endler, Karen L. Cheney

**Affiliations:** ^1^ Marine Sensory Ecology Group, The University of Queensland, School of Biological Sciences, Brisbane, QLD 4072, Australia; ^2^ Centre for Integrative Ecology, School of Life and Environmental Sciences, Deakin University, Geelong, Victoria 3216, Australia; ^3^ Zoology and Ecology Tropical Environments Sciences, College of Science and Engineering, James Cook University, Smithfield Cairns, QLD 4878, Australia

**Keywords:** aposematism, crypsis, visual modelling, secondary defences, predator psychology, signalling honesty

## Abstract

Aposematic signals visually advertise underlying anti-predatory defences in many species. They should be detectable (e.g. contrasting against the background) and bold (e.g. using internal pattern contrast) to enhance predator recognition, learning and memorization. However, the signalling function of aposematic colour patterns may be distance-dependent: signals may be undetectable from a distance to reduce increased attacks from naïve predators but bold when viewed up close. Using quantitative colour pattern analysis, we quantified the chromatic and achromatic detectability and boldness of colour patterns in 13 nudibranch species with variable strength of chemical defences in terms of unpalatability and toxicity, approximating the visual perception of a triggerfish (*Rhinecanthus aculeatus*) across a predation sequence (detection to subjugation). When viewed from an ecologically relevant distance of 30 cm, there were no differences in detectability and boldness between well-defended and undefended species. However, when viewed at closer distances (less than 30 cm), well-defended species were more detectable and bolder than undefended species. As distance increased, detectability decreased more significantly than boldness for defended species. For undefended species, boldness and detectability remained comparatively consistent, regardless of viewing distance. We provide evidence for distance-dependent signalling in aposematic nudibranchs and highlight the importance of distinguishing signal detectability from boldness in studies of aposematism.

## 1. Introduction

Conspicuous colour patterns displayed by aposematic species educate predators about underlying defences during prey encounters [1,2] or via eavesdropping (e.g. [3]). Aposematic colour patterns should be readily detectable against their visual background, and detectability is crucial to the initial evolution of aposematism [4,5]. Most aposematic signals are also bold, defined as being bright and colourful, which includes exhibiting high internal colour and luminance contrast [6,7] and striking colour pattern geometry [1,2,8,9]. This enhances the formation and maintenance of predator avoidance behaviour [10]. Theoretically, aposematic animal colours and patterns should be most efficient when they are both easily detected and bold [4].

However, it is essential to differentiate between detectability and boldness to understand the appearance of a visual signal in the context of its background and the pattern itself. Evidence for and against the relative contribution of each to signal quality and efficacy is mixed [11,12]. For example, Sillén-Tullberg [13] demonstrated that chicks (*Gallus gallus domesticus*) learned to avoid coloured artificial prey independent of background contrast. By contrast, Aronsson and Gamberale-Stille [14] failed to find an effect of internal patterning on the strength of avoidance behaviour in chicks. A key benefit of increased detectability in aposematic species may be to reduce predator recognition errors by allowing more time for accurate decision-making [15]. However, Gamberale-Stille *et al.* [16] demonstrated that increased detectability in chickens (*Gallus gallus domesticus*) did not improve the survival of aposematic prey. In fact, if detectable, aposematic species are attacked more frequently by naïve predators or those capable and willing to tolerate secondary defences (e.g. [17,18]). Detectability can therefore be a potential handicap of bold aposematic signals rather than a fitness benefit [4,19–23] and could be under conflicting selection pressures for and against increased levels of conspicuousness.

Aposematic species may benefit from distance-dependent signalling [21,24], which would allow optimal levels of detectability while maintaining high signal boldness. This shift in colour pattern functionality could reduce the detectability of bold colour patterns from a distance while enabling predator deterrence up close. Multiple theoretical and empirical studies support this principle (e.g. [25–31]). For example, Barnett & Cuthill [22] demonstrated that, when searched for by human observers, camouflaged stimuli with boldly contrasting markings were detected at the same distance compared to camouflaged stimuli without bold markings. This combination of colour pattern boldness and camouflage was later confirmed using artificial stimuli and avian predators [32–34].

Distance-dependent signalling can be investigated by considering the visual perception of a signal in its ecological context, including the spatial acuity of a visual system and the distance from which it is observed [21,35,36]. As viewing distances increase, the perception of higher spatial frequencies in visual backgrounds and animal colour patterns is lost. Smaller-scale colour pattern elements blur together, leading to the additive blending of colour pattern elements [37]. How, and if, such blending happens is influenced by the size, shape and colour of individual colour pattern elements [38]. As a result, individual or multiple colour pattern design elements can be subject to single or multiple selective pressures depending on the contextual relevance of their perception [39,40]. Therefore, despite being influenced by the loss of spatial detail as viewing distances increase, colour pattern boldness and detectability in aposematic animals may not be subject to equal selective pressures at varying viewing distances (e.g. [34]).

In the predator sequence, prey detection precedes prey identification and subsequent decisions by predators to further engage with prey [41,42]. Therefore, context-specific signal processing is used by predators as they proceed from detection to discrimination and subsequent attack [12,39,43]. The relative importance of primary and secondary defences likely shifts along such a predation sequence (see [44] for review). For example, visual defences relying on the avoidance of detection precede bold deimatic displays by threatened prey warning a predator of underlying secondary defences (see [44,45] for reviews). However, it is unknown how detectability and boldness change along an escalating predation sequence in the context of permanently displayed warning signals. Few studies have differentiated between the detectability and boldness of aposematic animals (but see [46]) and done so at different viewing distances while considering the physiological limitations of ecologically relevant observers (but see [26]). This is partly due to the challenge of capturing spatiochromatic properties of complex visual backgrounds according to observer-specific physiological limitations, such as spatial acuity and chromatic/achromatic contrast perception under natural illumination.

**Figure 1.**
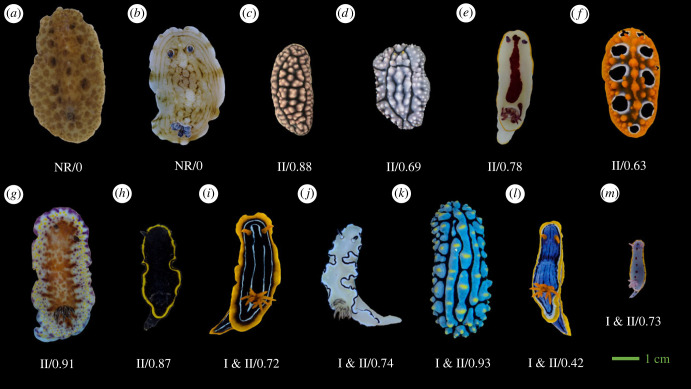
Representative images of species used in this study grouped by their chemical defences (scaled approximately according to size): (*a*) *Discodoris* sp*.;* (*b*) *Aphelodoris varia;* (*c*) *Phyllidiella pustulosa;* (*d*) *Phyllidia elegans;* (*e*) *Goniobranchus splendidus;* (*f*) *Phyllidia ocellata;* (*g*) *Goniobranchus collingwoodi;* (*h*) *Glossodoris vespa;* (*i*) *Chromodoris kuiteri;* (*J*) *Doriprismatica atromarginata;* (*k*) *Phyllidia varicosa;* (*l*) *Chromodoris elisabethina;* (*m*) *Hypselodoris bennetti*. The level of chemical defence is provided as values below each image determined from toxicity and unpalatability assays in Winters *et al*. [49,56]. The first value indicates the class of chemical defence: 'NR' indicates no response in assays and therefore nudibranch species that have limited or no chemical defences; class 'II' indicates highly unpalatable but weakly toxic species; and class 'I & II' indicates species that are highly unpalatable and highly toxic. The second value is ED_50_ data from unpalatability feeding assays with Palaemon shrimp. ED_50_ refers to the median effective dose at which 50% of pellets are rejected, presented here as 1 – ED_50_ values normalized to range from 0–1, where 1 indicates the least palatable species.

In this study, we investigated the detectability and boldness of defensive animal coloration in 13 species of nudibranch molluscs with differing chemical defences (figure 1). Nudibranch molluscs display a stunning diversity of defensive coloration and secondary defences, and are a valuable model system in the study of the ecology and evolution of aposematic signals [46–49]. We used quantitative colour pattern analysis (QCPA) [50] and considered the distance from which nudibranchs would be perceived by an ecologically relevant observer, a triggerfish (*Rhinecanthus aculeatus*). We hypothesized that when viewed from larger distances, the detectability of nudibranchs against their backgrounds should be low for all species, irrespective of their bold colour patterns or the strength of chemical defences. However, when viewed up close, we hypothesized that species with strong chemical defences would show significantly greater visual contrasts against their backgrounds than undefended species. Lastly, we expected an unequal change in the relationship of boldness and detectability between chemically defended and undefended species across different viewing distances.

## 2. Material and methods

### (a) Image collection

We took calibrated digital images of 13 Dorid nudibranch species (*n* = 226 individuals): *Aphelodoris varia* (*n* = 24), *Chromodoris elisabethina* (*n* = 21), *Chromodoris kuiteri* (*n* = 17), *Discodoris* sp*.* (*n* = 15), *Doriprismatica atromarginata* (*n* = 27), *Glossodoris vespa* (*n* = 15), *Goniobranchus collingwoodi* (*n* = 15), *Goniobranchus splendidus* (*n* = 25), *Hypselodoris bennetti* (*n* = 10)*, Phyllidia elegans* (*n* = 8)*, Phyllidia ocellata* (*n* = 23), *Phyllidia varicosa* (*n* = 8) and *Phyllidiella pustulosa* (*n* = 18). Species were visually identified using various taxonomic books [51–53]. We grouped individuals that visually resembled *Sebadoris fragilis* and were found in the same locations as *Discodoris* sp.; however, these individuals could be a mixture of *Sebadoris fragilis, Thayuva lilacina, Jorunna pantheris* and perhaps other undescribed species that cannot be identified without molecular sequencing.

Fieldwork was conducted on SCUBA between October 2017 and July 2021 at dive sites along the east coast of Australia: Sunshine Coast (SE Queensland), Gold Coast (SE Queensland) and Nelson Bay (New South Wales). Individual nudibranchs were located and photographed underwater at depths of 2–18 m against their natural habitat using a calibrated digital Olympus EPL-5 with a 60 mm macro lens in an Olympus PT-EP10 underwater housing. We used white LED illumination from a combination of VK6r and PV62 Scubalamp video lights for illumination (as per [50]). All images were taken at roughly a 90° angle (top–down) relative to the animal and its background, with the animals in a naturally stretched-out, straight, forward-moving position (figure 1). All pictures were taken with a colour and size standard placed next to the animal (see the Supplement of [47]). As the images were taken with a prime lens, the distance from which photographs were taken varied as a function of nudibranch size: larger animals were photographed from further away (approx. 60 cm) compared to smaller ones (approx. 30 cm). At such close distances, scattering by water was assumed to be negligible and thus did not impact image analysis.

### (b) Aposematism and chemical defences

We quantified chemical defences using previously published measures of toxicity (causing injury and harm) and deterrency (unpalatability) [48]. These measures of defence are only sometimes correlated [49,54,55] and thus should be disentangled in the aposematism literature. First, we used both measures of toxicity and unpalatability using the classification from [49]. Species belonged to the following three classes: no response ‘NR’, nudibranch species that have limited or no chemical defences; ‘Class II’, species that are only weakly toxic but highly unpalatable; ‘Class I & II’, highly toxic and highly unpalatable. *Glossodoris vespa* was classified as Class II from assay data reported in [56]. In [56], only one species belonged to Class I (highly toxic and weakly unpalatable) and we did not have sufficient colour pattern data for this species. Our second measure was unpalatability alone. Here, we ranked our study species from 0 to 1, according to the average ED_50_ (Effective Dose, 50%) response in *Palaemon* shrimp feeding assays reported in [49,56], calculated as 1 − ED_50_ and normalized so that 1 represented the most unpalatable species (figure 1).

Except for *Discodoris* sp*.* and *Aphelodoris varia*, we consider these species to be aposematic as they harbour potent chemical defences and display bold colour signals ([49,56], figure 1). *Aphelodoris varia* has no known chemical defences [49] and likely relies on crypsis via background matching for their primary defence. *Sebadoris fragilis* has no known chemical defences [49] and we therefore classified *Discodoris* sp*.* as non-toxic and palatable.

### (c) Quantification of detectability and boldness

Image analysis was conducted with visual modelling parameter choices as per [38] at the following viewing distances: 2 cm, 5 cm, 10 cm and 30 cm. At distances beyond 30 cm, the majority of small nudibranchs and much of the internal patterning in larger individuals are unlikely to be visible to a triggerfish (*R. aculeatus*) due to its spatial acuity of about three cycles per degree (cpd) [57].

To quantify detectability (i.e. a measure of background matching), we calculated the absolute difference of the abundance-weighted coefficient of variation of achromatic (*Lum.CoV*) and chromatic (*Col.CoV*) local edge contrast (LEIA, measured in Δ*S*) for each animal relative to that of its respective visual background. Thus, by considering the difference in the overall appearance of an animal by itself relative to its immediate background, we specifically quantify background matching. The coefficient of variation of chromatic and achromatic boundary contrast between colour pattern elements has previously been shown to indicate mate choices in guppies (*Poecilia reticulata*) [58,59]. Instead of using the boundary strength analysis (BSA) [60], we make use of the non-parametric approach of LEIA and its ability to consider colour pattern contrast at roughly the scale of an edge-detecting receptive field of a triggerfish [61]. Unlike BSA, LEIA does not rely on image segmentation. After applying Gaussian acuity modelling, we use receptor noise limited (RNL) ranked filtering [50] with settings chosen as per [38].

The RNL model [62] allows us to estimate the perceived colour and luminance contrast of non-human observers and, in addition to RNL ranked filtering, is used to describe the contrast of edges within each animal and its corresponding natural background. This contrast measure is expressed as *Δ*S and reflects the distance between two contrasting points in the RNL colour space [63]. We used the mean LEIA contrast across the vertical, horizontal and diagonal filter axis to describe the colour and luminance contrast at each location in the image. For a detailed description of LEIA parameters, see [50].

To quantify boldness, we used *Lum.CoV* and *Col.CoV* of each animal without considering their respective visual backgrounds, as we define the boldness of an aposematic signal as the strength of the signal once prey detection has taken place. High values for each pattern statistic indicate the presence of highly contrasting colour pattern elements, whereas low values indicate a pattern with no or weakly contrasting colour pattern elements.

### (d) Statistical analysis

All colour pattern statistics were normalized to range from 0 to 1 using the ‘range’ argument of the PreProcess function (caret package, [64], v. 6.0-90) using R Software ([65], v. 4.1.2). When we considered differences between species without considering the class of their chemical defences, detectability and boldness data did not meet the assumptions of normality due to large variation in the number of individuals and individual trait data, so we used a Kruskal-Wallis test [66]. This analysis was performed using the *stats* package and *post hoc* analyses comparing individuals was done using Dunn tests [67] with a Bonferroni correction [68] in the *FSA* package ([69],v. 0.9.3).

When considering differences between species with their class of chemical defence at different viewing distances, we applied a linear mixed effect (lme) model using the lme4 ([70], v. 1.1-28) and lmerTest ([71], v. 3.1-3) packages and type III ANOVA tables using Saterwaite's method in the stats package after applying a square-root transform to the left-skewed data. The meeting of assumptions was assessed for each model fit. We further ran independent lme models for chemical defence and viewing distance. Species was treated as a random effect for all lme models.

To determine the relationship between unpalatability data and colour pattern statistics, we used a Pearson product-moment correlation (*R*) in the stats package ([65], v.4.1.2). Detectability and boldness were measured as the median species value of *Lum.CoV* and *Col.CoV* at each viewing distance. For this analysis, we applied an ordered quantile normalization to the raw data using the bestNormalize package ([72], v.1.8.2) to ensure normality throughout the dataset. The distance-dependent change in the correlation between unpalatability and visual defences was then assessed by fitting linear regressions to the obtained Pearson product–moment correlations.

## 3. Results

### (a) Detectability

We first considered differences in detectability between species (i.e. the difference between each animal and its background) at different distances without considering chemical defences. We found significant differences in achromatic detectability (*Lum.CoV*) among species at all viewing distances (Kruskal-Wallis 2 cm: $\chi_{\left(12\right)}^{2}=126.35,$* p* < 0.001; 5 cm: $\chi_{\left(12\right)}^{2}=111.44,$ *p* < 0.001; 10 cm: $\chi_{\left(12\right)}^{2}=75.389,$ *p* < 0.001; 30 cm: $\chi_{\left(12\right)}^{2}=24.94,$ *p* = 0.015). However, only one species remained significantly more detectable than others at 30 cm, with *H. bennetti* being more detectable than *G. vespa* (Dunn test: *p*_adj_ = 0.021, electronic supplementary material, table S1). Similarly, there were significant differences in chromatic detectability among species at close viewing distances (Kruskal-Wallis 2 cm: $\chi_{\left(12\right)}^{2}=95.23,$ *p* < 0.001; 5 cm: $\chi_{\left(12\right)}^{2}=97.69,$ *p* < 0.001; 10 cm: $\chi_{\left(12\right)}^{2}=76.81,$ *p* < 0.001) but not at 30 cm (Kruskal-Wallis: $\chi_{\left(12\right)}^{2}=16.62,$ *p* = 0.164, electronic supplementary material, table S2.)

**Figure 2.**
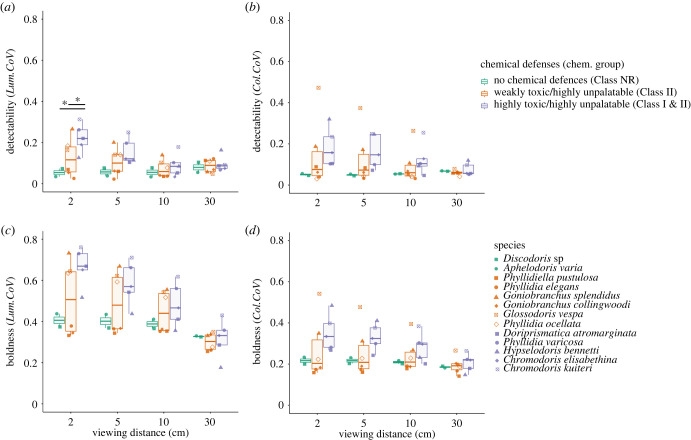
Relative detectability and boldness of 13 dorid nudibranch species. The strength of chemical defences is indicated by colours relative to the class of chemical defence. (*a*) Species median detectability quantified as the abundance-weighted coefficient of variation of achromatic local edge contrast (*Lum.CoV*) measured relative to the natural background of every individual. (*b*) Species median detectability quantified as the abundance-weighted coefficient of variation of chromatic local edge contrast (*Col.CoV*) measured relative to the natural background of every individual. (*c*) Species median achromatic pattern boldness measured as the abundance weighted coefficient of variation of each animal's achromatic local edge contrast (*Lum.CoV*). (*d*) Species median chromatic pattern boldness measured as the abundance-weighted coefficient of variation of each animal's chromatic local edge contrast (*Col.CoV*).

When considering the class of chemical defence, species without chemical defences (NR) had higher achromatic detectability (*Lum.CoV*) at larger viewing distances (30 cm) than up close (less than 30 cm; *F*_4,189_ = 6.27, *p* < 0.001). By contrast, chemically defended species were less detectable when viewed from a distance, with the most defended species (Class I and II) displaying the largest differences between viewing distances (weakly toxic/highly unpalatable (Class II): *F*_4,510_ = 12.31, *p* < 0.001; highly toxic/highly unpalatable (Class I & II): *F*_4,406_ = 31.38, *p* < 0.001; figure 2*a*).

For species without chemical defences (NR), there was no difference in chromatic detectability (*Col.CoV*) among viewing distances (*F*_4,190_ = 0.50, *p* = 0.734). By contrast, chemically defended species showed a significant reduction in chromatic detectability with increased viewing distances, and again this was most pronounced in the most defended species (weakly toxic/highly unpalatable (Class II): *F*_4,510_ = 12.24, *p* < 0.001; highly toxic/highly unpalatable (Class I & II): *F*_4,509.98_ = 16.13, *p* < 0.001; figure 2*b*).

There was no significant difference in achromatic or chromatic detectability among chemical defence groups at viewing distances of 5, 10 or 30 cm (achromatic detectability [*Lum.CoV*]: 5 cm, *F*_2,9.94_ = 2.07, *p* = 0.178; 10 cm, *F*_2,10.17_ = 0.27, *p* = 0.771; 30 cm, *F*_2,8.58_ = 0.07, *p* = 0.937; chromatic detectability [*Col.CoV* ]: 2 cm, *F*_2,9.82_ = 1.09, *p* = 0.374; 5 cm, *F*_2,9.88_ = 1.03, *p* = 0.391; 10 cm, *F*_2,9.95_ = 0.75, *p* = 0.498; 30 cm: *F*_2,8.68_ = 0.22, *p* = 0.807). However, at 2 cm, achromatic detectability of highly defended species (Class I & II) were more detectable than species without chemical defences (Class NR) or unpalatable ones (Class II; *F*_2,9.8_ = 4.26, *p* = 0.047).

**Figure 3.**
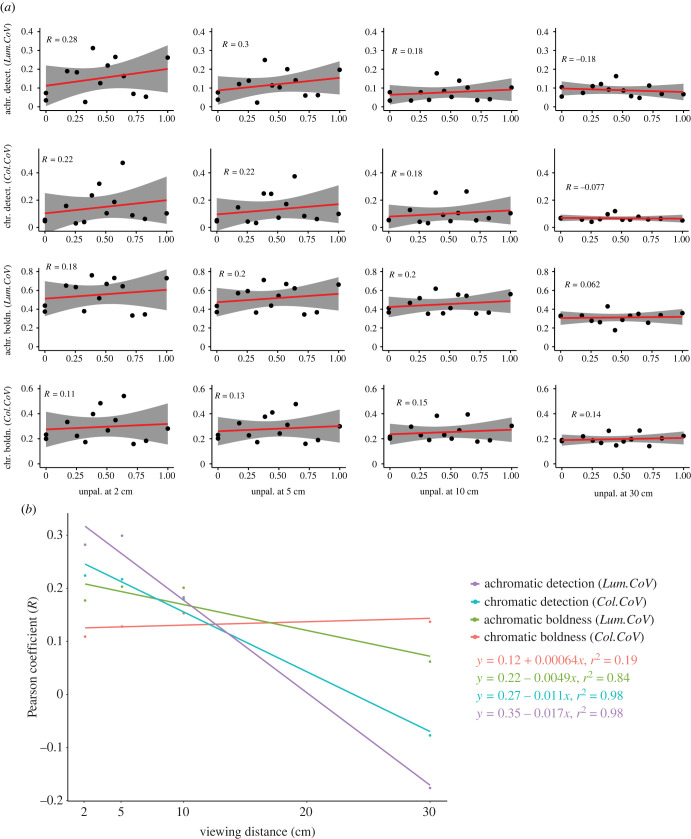
Summary of the correlation of the median chromatic (chr., *Col.CoV*) and achromatic (achr., *Lum.CoV*) boldness (boldn.) and detectability (detect.) of each species with the normalized strength of chemical defences (1 – ED_50_) across different viewing distances. (*a*) Individual Pearson product–moment correlations (*R*) for each viewing distance and colour pattern statistic. Grey shading shows 95% confidence intervals; (*b*) Linear regressions fitted to the Pearson product–moment correlations (*R*) show variable distance-dependent decreases in the correlation between colour pattern statistics.

When considering chemical defences on a continuous scale using unpalatability data, there was a weak positive relationship between unpalatability and both achromatic (*R* = 0.18–0.28) and chromatic detectability (*R* = 0.18–0.22) at close viewing distances (2 cm–10 cm). By contrast, no or weakly negative relationships (*R* = −0.077 – −0.18) were observed at larger viewing distances (30 cm, figure 3*a*). Both achromatic (*r^2^* = 0.98, *F*_1,2_ = 117.7, *p* = 0.008) and chromatic (*r ^2^*= 0.98, *F*_1,2_ = 110, *p* = 0.009) detectability showed a significant reduction in their relationship with unpalatability over increasing viewing distances (figure 3*b*).

### (b) Boldness

When we considered differences in boldness (i.e. analysing each animal without its background) without considering chemical defences, we found significant differences in achromatic boldness among species at all viewing distances (Kruskal-Wallis 2 cm: $\chi_{\left(12\right)}^{2}=159.94,$ *p* < 0.001; 5 cm: $\chi_{\left(12\right)}^{2}=161.11,$ *p* < 0.001; 10 cm: $\chi_{\left(12\right)}^{2}=148.26,$ *p* < 0.001; 30 cm: $\chi_{\left(12\right)}^{2}=74.27,$ *p* < 0.001). At 30 cm, significant differences between multiple species remained (figure 2*c*; electronic supplementary material, table S3). Similarly, there were significant differences in chromatic boldness among species across all viewing distances (Kruskal-Wallis 2 cm: $\chi_{\left(12\right)}^{2}=165.46,$ *p* < 0.001; 5 cm: $\chi_{\left(12\right)}^{2}=163.55,$ *p* < 0.001; 10 cm: $\chi_{\left(12\right)}^{2}=147.8,$ *p* < 0.001, 30 cm: $\chi_{\left(12\right)}^{2}=88.89,$ *p* < 0.001; electronic supplementary material, table S4).

When considering the class of chemical defence, species with and without chemical defences showed decreased achromatic boldness at larger viewing distances, with highly defended species displaying the largest differences between distances (undefended (Class NR): *F*_4,189_ = 38.34, *p* < 0.001; weakly toxic/highly unpalatable (Class II): *F*_4,510.05_ = 244.74, *p* < 0.001; highly toxic/highly unpalatable (Class I & II): *F*_4,406_ = 324.83, *p* < 0.001; figure 2).

Similarly, species with and without chemical defences showed decreased chromatic boldness at larger viewing distances, with highly defended species displaying the largest differences between distances (undefended (Class NR): *F*_4,189_ = 6.27, *p* < 0.001; weakly toxic/highly unpalatable (Class II): *F*_4,510_ = 12.31, *p* < 0.001; highly toxic/highly unpalatable (Class I & II): *F*_4,406_ = 31.38, *p* < 0.001; figure 2*d*).

There were no significant differences in either achromatic (*Lum.CoV*, 2 cm: *F*_2,9.95_ = 2.43, *p* = 0.139, 5 cm: *F*_2,9.92_ = 1.33, *p* = 0.309, 10 cm: *F*_2,9.87_ = 0.34, *p* = 0.721, 30 cm: *F*_2,9.48_ = 0.19, *p* = 0.828) or chromatic boldness (*Col.CoV*, 2 cm: *F*_2,9.95_ = 1.08, *p* = 0.375, 5 cm: *F*_2,9.97_ = 1.28, *p* = 0.320, 10 cm: *F*_2,9.80_ = 0.70, *p* = 0.519, 30 cm: *F*_2,9.77_ = 0.05, *p* = 0.947) between chemical defence groups at either viewing distance.

When considering chemical defences on a continuous scale using unpalatability data, both achromatic (*R* = 0.06–0.20) and chromatic boldness (*R* = 0.11–0.15) showed a weak positive relationship with unpalatability at all distances (2 cm–30 cm, figure 3*a*). However, neither achromatic (*r^2^* = 0.84, *F*_1,2_ = 10.38, *p* = 0.084) nor chromatic (*r^2^* = 0.19, *F*_1,2_ = 0.48, *p* = 0.560) boldness showed a significant reduction in their relationship with unpalatability over increasing viewing distances (figure 3*b*).

## 4. Discussion

Our study demonstrates that the appearance of nudibranch colour patterns, both in the context of natural backgrounds (detectability) or the colour pattern alone (boldness), depends on the distance from which an animal is viewed. For highly defended nudibranchs, both achromatic and chromatic detectability and boldness significantly reduced as viewing distance increased. By contrast, the detectability and boldness of apparently undefended species remained relatively consistent over viewing distances. Our results support predictions [21,24] and empirical findings [e.g. 22] suggesting that colour patterns with bold markings can be camouflaged at a distance, at a similar level to camouflaged species using background matching.

We first considered animal appearance across viewing distances, irrespective of underlying chemical defences. We found significant differences in chromatic and achromatic detectability between species at 2–10 cm, but little variation between species at 30 cm. However, for achromatic and chromatic boldness, significant differences remained among species at 30 cm. This indicates that some species, including those that are well-defended, may appear bold at larger viewing distances where the likelihood of detection is reduced but still possible. Using animals observed in their natural habitat, our findings support the idea that detectability and signalling function of defensive coloration can be differentially influenced by viewing distance, and that a reduction in detectability does not necessarily mean a reduction in signal quality [31,34].

We then investigated the detectability and boldness of species considering the strength of underlying chemical defences. Aposematic species should balance the costs and benefits of increased detectability induced by bold coloration [21,31,34]. Indeed, increased detectability and boldness at close viewing distances should enhance aposematic signals; however, aposematic species may likely benefit from being camouflaged at a distance [21,24]. Using our categorical measures of chemical defences, we found that species that were both toxic and unpalatable were more detectable at close viewing distances compared to unpalatable (but non-toxic) species or those lacking chemical defences (figure 2*a*). At 30 cm, we did not find significant differences in detectability between species with or without chemical defences (figure 2), which supports the assumption that both aposematic and cryptic species may profit from being camouflaged at larger viewing distances. Interestingly, detectability for undefended species was slightly greater at 30 cm compared to smaller distances (figure 2). This is likely due to the non-uniform differential blending of colours and patterns within the animals and their respective backgrounds, leading to a decrease in background matching at larger viewing distances. For boldness, variations in chromatic and achromatic measures were present between defended and undefended species (figure 2; electronic supplementary material, table S3 and S4); however, these differences were insignificant.

When considering chemical defences on a continuous scale (unpalatability), our results demonstrated that there was a sharper decline in detectability compared to boldness in chemically defended nudibranchs as viewing distance increased (figure 3). This finding aligns with predictions from the literature [31,34]. Specifically, both chromatic and achromatic detectability significantly decreased in unpalatable species. We did not observe a significant decrease in either chromatic or achromatic boldness in unpalatable species. Interestingly, highly unpalatable species are no longer more detectable than undefended species at around 10 cm (figure 3). At this distance, many of the more intricate colour pattern details are no longer visible to triggerfish, possibly making many animals more similar in appearance. Such distance-dependent effects on the perception of colour patterns could be crucial in the ecology and evolution of mimicry systems [73]. For example, the bold, outline-enhancing yellow rims displayed by some species in this study will likely be more detectable at a distance than the finer internal patterning. The loss of fine spatiochromatic detail at a distance could explain the presence and constancy of such outline-enhancing yellow rims [9] found on many aposematic nudibranch species and their putative mimics [40]. Indeed, the inability of ecologically relevant observers to resolve finer detail in animal coloration has been suggested to contribute to the evolution of imperfect mimicry (e.g. [73]).

Perceptual mechanisms underlying prey detection, identification and assessment are complex and remain insufficiently quantified in studies of visual ecology [38,44,50,74]. In this study, we quantified detectability and boldness using a pre-determined selection of image statistics based on existing literature, as per other studies in the field of visual ecology (e.g. [75,76]), rather than considering the entire colour pattern space available in QCPA. To further guide parameter choices, we must identify relevant visual features and modelling parameters in different behavioural contexts in future studies [38,74]. Furthermore, our study looks at relative differences of a specific colour pattern metric in a normalized dataset. As with most (if not all) colour pattern descriptors in the literature, we have yet to learn how absolute differences in these metrics relate to actual biological effect size for this particular context beyond literature-based assumptions (see [38,50,77] for discussion).

Furthermore, animal coloration and, therefore, colour patterns often serve more than just one function, thus often making them complex phenotypes resulting from the interplay of various selective forces (see [1,50,78,79] for discussion). In addition to neutral selection, this includes adaptive purposes of visual signalling other than for defence against visual predators, such as territorial and sexual signalling (e.g. [80]). It also includes purposes unrelated to vision, such as thermoregulation (e.g. [81,82]), and physical properties of pigmentation, such as abrasion resistance and pathogen defence (see [79] for discussion). This cumulative add-mixture of potential selective pressures acting on observed phenotypes makes it challenging to pinpoint causal relationships underlying the ecology and evolution of defensive animal coloration in even the best-understood animal systems used to study aposematic coloration. Nudibranch molluscs are particularly interesting as their diversity in visual defences is likely less confounded by various selective pressures. Most importantly, nudibranchs are aquatic hermaphrodites and lack visual abilities beyond mere phototaxis [83]. Therefore, nudibranchs provide a highly informative system for observing broadly generalizable causal relationships between prey morphology and visual predation, including those presented in this study.

Notably, our results show that the definition and quantification of primary and secondary defences influence the strength of correlations found between them (figures 2 and 3). For example, when considering unpalatability on a continuous scale, we found that the correlation between detectability and the strength of chemical defences decreases significantly with increasing viewing distances, whereas this does not happen for boldness (figure 3). However, when looking at chemical defences defined as categories, we find significant decreases in boldness for chemically defended species with increasing viewing distances (figure 2). Furthermore, when considering categories of chemical defences, the effect size for the decrease in achromatic boldness with increasing viewing distance is much larger than that for the decrease in chromatic boldness, highlighting the importance of considering multiple measures of colour pattern appearance.

It is essential to acknowledge that the quantification of colour patterns according to animal vision is constantly advancing (see [50] for discussion). Therefore, how colour pattern detectability and boldness are considered in our study is guided by modelling choices on colour, luminance, spatial and spatiochromatic contrast perception according to a specific selection of methods and literature. However, independently of how physiologically defined sensory limitations of ecologically relevant observers are considered, they remain relevant for all visually guided animal behaviour. For example, the blurring and fusion of spatiochromatic information with increasing viewing distances will occur for all species in all habitats due to physical optics [38,50,84]. Thus, the importance of distinguishing between signal detectability and signal boldness in any predator–prey system is not guided by the intricacies of how their characterization is achieved but simply by acknowledging the possibility of considering them as distinct perceptual properties.

In summary, our findings highlight that when investigating aposematic signals, it is important to distinguish between signal detectability and boldness in the context of ecologically relevant viewing distances, and the term ‘conspicuousness’ should therefore be well-defined. We suggest that many aposematic nudibranchs are cryptic when viewed at larger viewing distances, and levels of detectability are similar to undefended species that use background matching. However, differential, distance-dependent selection on detectability and colour pattern boldness likely also applies to other forms of visual signalling in prey animals, such as sexual or territorial signalling, suggesting a significant area of investigation for future studies.

## Data Availability

The data are available on UQ e space: https://doi.org/10.48610/03c56dd [85] and in the electronic supplementary material [86].
